# The Moderating Impact of Attending Services on the Effect of Older Adults’ Discrimination Experiences on Loneliness

**DOI:** 10.1093/geroni/igaa057.1035

**Published:** 2020-12-16

**Authors:** Dahee Kim, Kyuho Lee

**Affiliations:** 1 Iowa State University, Ames, Iowa, United States; 2 Daegu University, Gyeongsan, Kyongsang-bukto, Republic of Korea

## Abstract

Research has shown that perceived discriminations impact physical and mental health in later life. Discrimination experiences could make older adults consider themselves as a social misfit and decrease their social interactions, which finally increases their loneliness. Religious behaviors has been reported as a key factor of a lower sense of isolation. Considering that religious behaviors provide opportunities to engage in more extensive social networks and have supportive social ties with community members, attending religious services might decrease the impact of older adults’ perceived discrimination on loneliness. The current research aims to examine the moderating role of religious services attendance in the association between older adults’ perceived discrimination and loneliness. We used data of 4,488 adults aged 50 to 80 (M=66.27, SD=10.15) from the Health and Retirement Study (HRS) collected in 2012 and 2014. Linear regression analysis was performed to investigate whether older adults’ religious service attendance might decrease the impact of their perceived discriminations in daily life on the level of loneliness. The results indicated that more perceived discriminations older adults face on a daily basis were significantly associated with higher levels of loneliness. However, participants who frequently attended religious services showed a lower impact of perceived discriminations on their loneliness. These findings highlight the positive effects of engaging in religious activities on discriminated older adults’ social well-being. These findings also emphasize the role of the religious community as a social resource for socially marginalized older adults.

